# The impact of socio-cultural values on autistic women: An interpretative phenomenological analysis

**DOI:** 10.1177/13623613211037896

**Published:** 2021-08-13

**Authors:** Stella Mo, Nina Viljoen, Shivani Sharma

**Affiliations:** 1 University of Hertfordshire, UK; 2 Hertfordshire Partnership University NHS Foundation Trust, UK

**Keywords:** autism, culture, identity, interpretative phenomenological analysis, women

## Abstract

It is well recognised that culture plays an important role in how people experience the world. However, there is limited knowledge on the impact of socio-cultural norms and values on the lives of autistic women. This qualitative study used individual semi-structured interviews to explore how eight cis-gendered autistic women, without co-occurring intellectual disabilities, describe dominant socio-cultural beliefs, values and norms and their influence on their own sense of self. Findings elucidated three interdependent themes related to the ‘pervasive influence of cultural values’, ‘individualisation as an autistic woman’ and ‘social connectivity’. Autistic women described how they experienced their environments and made choices about their place within the dominant culture and the impact of these decisions on their identities and experience. The findings of this study have implications for the continued need to shift societal and clinical attitudes towards understanding and appreciating diversity among autistic women.

**Lay abstract:**

Autistic women with average or above intellectual abilities are often overlooked clinically or identified at older ages compared to autistic males. Their experiences can provide insight into the socio-cultural factors that impact on how they develop and are seen by others. This study asked autistic women to describe the culture around them and explore how this has influenced their lived experiences. Individual semi-structured interviews were conducted with eight autistic women without a co-occurring diagnosis of intellectual disabilities. These were used for interpretative phenomenological analysis. Overall, we found three closely connected themes on the pervasive influence of cultural values on autistic women, how autistic women define themselves and the importance of connecting with society. These findings suggest that dominant cultural beliefs, values and norms effect how autistic women are recognised by others and develop their sense of self. Broadening how people think about autistic women in society and clinically may benefit how we identify and support autistic women.

## Introduction

Culture encompasses the implicit and explicit beliefs, values, attitudes and behaviours of a social group that are passed on through relationships between people (Beldo, 2010; Hudelson, 2004). Culture exists as a dynamic process of mutual influence between individuals and their contexts (Hudelson, 2004; Krause, 1995). Hofstede et al. (2010) emphasised that culture both impacts behaviour as well as its interpretation. Therefore, considering the cultural contexts of autistic people is important, since autism is, in part, a socially constructed phenomenon (Leveto, 2018; Nadesan, 2005; Runswick-Cole et al., 2016).

Autism is diagnosed by a set of behaviours determined by their divergence from social norms; norms that are defined within social, clinical and research cultures (Davies, 2016; Timimi & McCabe, 2016). Although culture impacts the day-to-day experiences of autistic people and their families, Milton (2013) drew attention to the lack of autism research at the micro-sociological level. Furthermore, the prevailing focus within autism research on biology, brain and cognition means that societal issues have been largely neglected (Pellicano et al., 2013). This has been met by an increase in qualitative research with firsthand accounts on critical issues that exemplify how culture contributes to the experience of autism in different settings, for example, diagnosis and access to care (Alqahtani, 2012; Gordillo et al., 2020; Legg & Tickle, 2019).

Notably, clinical and research cultures have facilitated a biased understanding of autism (Kreiser & White, 2014; Young et al., 2018). This stems from the high male-to-female ratio, which has been estimated at 4:1 (Fombonne, 2003, 2009), reducing to 3:1 when taking into account methodological merit and active case-ascertainment approaches (Loomes et al., 2017). Although sex differences in autism have been studied extensively, the fact that more males are diagnosed (Fombonne, 2009) has continued to perpetuate a male-centric understanding. This is supported by evidence that indicates a diagnostic bias against autistic girls without intellectual disabilities (IDs) (Dworzynski et al., 2012), and autistic girls with average or above intelligence (Kim et al., 2011); contributing to females being diagnosed at an older age (Begeer et al., 2013).

Although biological vulnerability has a role to play, gendered socialisation has also been considered to influence such disparities (Cheslack-Postava & Jordan-Young, 2012). A plethora of research has identified processes through which gender socialisation occurs from childhood and impacts identity and role formation (Carter, 2014; Stockard, 1999). Such gendered socialisation may affect the expression of autism, as well as interpretation of behaviours by professionals (Kreiser & White, 2014). For example, some studies have suggested that autistic females are subjected to higher expectations for engaging with social behaviour by their parents compared to autistic males (Holtmann et al., 2007; McLennan et al., 1993). Observations of autistic and non-autistic boys and girls in the playground also evidence that the female social landscape, which emphasises more joint engagement, leads to autistic girls camouflaging social difficulties from adults (Dean et al., 2017). These findings score the importance of attending to socio-cultural processes in facilitating a deeper understanding of autism.

Milton (2013) further highlighted how stigma can mediate the interaction between cultural perceptions of autism and the self-perception of autistic people. Autistic people tend to perceive the world differently to non-autistic people, and this can lead to a ‘double empathy problem’ where both parties experience difficulties empathising with each other (Milton, 2012). However, the dominant narrative in society situates the empathy deficit in autistic people, which can pathologise differences.

For autistic women, such stigmatisation may be intensified by the incongruence between expectations about appearance and behaviour associated with females (Gould & Ashton-Smith, 2011; Kreiser & White, 2014). For example, a narrative analysis of the lived experiences of seven autistic women demonstrated the heterogeneity of connection to gender-based stereotypes (Kanfiszer et al., 2017). The distress and negative self-evaluation felt from not being a ‘girly girl’ or having ‘maternal instincts’ was prominent in their sense of self (Kanfiszer et al., 2017). This exemplifies the pressure to fulfil cultural stereotypes of being ‘normal’ (Bargiela et al., 2016).

Paradoxically, many autistic women have also experienced misdiagnoses and been denied autistic identities due to stereotypes about autism (Bargiela et al., 2016; Tint & Weiss, 2018). This can hinder everyday experiences such as motherhood where women have described challenges following disclosure of autism to healthcare providers (Pohl et al., 2020). Motherhood is not inherently associated with stereotypes about autism (Gardner et al., 2016; Rogers et al., 2017).

The multiple influences on autistic women stemming from different cultural norms, values and attitudes are vast, and clearly evidenced in their own narratives of life experiences (Bargiela et al., 2016; Kanfiszer et al., 2017; Rogers et al., 2017; Tint & Weiss, 2018). Yet, research directly on the relationship that autistic women have with their socio-cultural contexts is lacking. The aim of this qualitative study was to delve deeper into how autistic women have made sense of their present and past experiences in the context of the culture in which they are embedded. This is to provide a richer and more inclusive understanding of the biopsychosocial context that shapes everyday life for autistic individuals.

## Method

### Overview

The study was based on Interpretative Phenomenological Analysis (IPA), which has been described as a useful qualitative approach for facilitating research with autistic individuals (Howard et al., 2019; MacLeod, 2019). This is because of IPA’s theoretical underpinnings which acknowledges the contexts of researchers and participants (Eatough & Smith, 2017) through a rigorously reflexive interpretation process (Larkin et al., 2006), and centres the perspectives of participants through an idiographic focus on meaning-making of experiences (Smith et al., 2009). Interviews were facilitated by the first author who was a trainee clinical psychologist with previous experience with autistic individuals. Reflexivity was essential to ensure that pre-conceived ideas and experiences were suspended (‘bracketed’) as much as possible (Larkin & Thompson, 2012).

### Participants and recruitment

Consistent with recommendations by Howard et al. (2019) on using IPA in autism research, homogeneity was attended to by focusing solely on cis-gendered autistic women based in England and without co-occurring ID. This recognised that (1) women who identify with a gender that is different to sex assigned at birth may have unique experiences and that (2) women based in other majority cultural settings may also have specific influences that impact their sense-making. Furthermore, we did not include women with ID as their experiences warrant exploration in their own right.

Eight autistic women participated, recruited through purposive sampling (Table 1). Seven women were recruited among staff and students from the first author’s higher education institution, and one woman was recruited from a charity supporting autistic adults. All participants were adults over 18 years old, with a formal diagnosis of autism (American Psychiatric Association, 2013; World Health Organization, 2018), without co-occurring ID. Although the age at both diagnosis and at the time of interview varied, this was considered integral to understanding how women have experienced their socio-cultural environments.

**Table 1.** Pseudonyms and demographic data of participants.

Participant	1	2	3	4	5	6	7	8
Pseudonym	Lola	Ambie	Marion	Trinity	Sarah	Lauren	Rebecca	Emma
Age	34	48	53	25	20	20	24	18
Age at diagnosis	30	40	50	9	16	12	22	16
Gender	Female	Female	Female	Female	Female	Female	Female	Female
Preferred terminology	Autistic woman	Autistic woman	Autistic woman	Autistic person	No preference	Woman with Autism	Autistic person/woman	Woman with Asperger’s syndrome/ASD
Ethnicity	White	White	White	White	White	Undisclosed	White	White
Education level	Undergraduate degree (in progress)	GCSEs	Doctorate (in progress)	Undergraduate degree (in progress)	Undergraduate degree (in progress)	Undergraduate degree (in progress)	Undergraduate degree (in progress)	Undergraduate degree (in progress)
Career field	Psychology	Poet	Health	Performance and production	Media and communications	Education	Engineering	Nursing
Religion	None	None	None	None	None	Christian	Agnostic	Christian
Family of origin’s religion	Catholic	None	None	None	None	Christian	None	Christian
Siblings	Young brother and sister	Two younger sisters	Older brother	Two younger sisters	None	An older sister and eight younger siblings	Younger sister	Younger brother and sister
Autism diagnosed in family	None	Niece and cousins	Son	None	Father	Sister	None	Cousin

ASD: autism spectrum disorder; GCSEs: General Certificate of Secondary Educations.

### Procedure

Semi-structured interviews were completed by the first author. Participants were provided with the following definition of culture: ‘The values, beliefs, attitudes, and behaviour of a particular social group or society’. The interview structure started with exploring culture (‘What kind of values, beliefs and attitudes did you grow up around?’), followed by life experiences (‘Could you tell me a bit about your life so far?’) and ended with reflecting on how cultural values have impacted their life experiences (How do you think some of the cultural values around you have made it harder/easier for you?). Prompting was kept to a minimum to prioritise the participant’s interpretation. This interview schedule was used flexibly to facilitate the sense-making process of the participant; for example, some participants found it easier to start with talking about their experiences and explorations of their culture were interweaved throughout. Interviews lasted between 60 and 139 min; average interview length was 90 min.

### Ethical considerations

All women provided informed consent to participate and permission to record interviews; these were anonymised at the point of transcription and pseudonyms used throughout.

To enhance accessibility, differences in social communication and perception were considered. Participants were provided opportunities to discuss queries or concerns about participation. Their preferred terminology for autism was used during interactions. Example adaptations included forwarding the interview schedule in advance and booking quieter interview spaces. All participants were offered face-to-face or remote interviews; all participants chose the former, but this was not possible for one participant and video-link was used instead.

Participants were routinely forwarded the participant information sheet, consent form and details (time, location) before the interview. With the exception of the video-link interview, two adjacent interview rooms were booked so that a private space was available to the participant before, during and after the interview to use as they wish (e.g. settle in). Participants were reminded that they could take breaks as needed, and it was emphasised that they did not have to talk about anything they did not want to. To support those who may benefit from processing information visually, the definition of culture and the interview questions were printed or made available on a screen.

### Analysis

All analyses were completed by the first author, which followed a number of cyclical stages aimed to create an idiographic focus on each participant (Smith et al., 2009). Interviews were transcribed verbatim to aid with immersion in the data. Exploratory thoughts were categorised into descriptive, linguistic and conceptual categories. These were then concentrated into emergent themes alongside the original transcript, and were further refined to three or four master themes using a range of strategies (e.g. abstraction). The above stages were repeated for each interview. The master themes from all the interviews were then collated to develop superordinate themes and subordinate themes. A research journal was used to aid reflexivity throughout the analysis. Peer examination was also important for providing rigour (Shenton, 2004). All authors met frequently throughout the analysis process to review the fit of the data with the themes. This included the first author revisiting and further refining analysis. Analyses were completed when there was consensus that the data reflected the main findings of the research.

### Community involvement

The study aimed to recruit autistic women without ID to consult on this research. Recruitment for consultation was attempted through a charity for autistic people that facilitated research participation. Unfortunately, no consultants were identified after an extended period of recruitment. The first author therefore consulted with professionals with research, clinical and personal experiences with autistic people. The interview process was also refined through participant feedback.

## Results

Three superordinate themes were identified and are detailed alongside subordinate themes in Table 2.

**Table 2.** Superordinate and subordinate themes.

Superordinate themes	Subordinate themes
Pervasive influence of cultural values	Power in the unsaid Obscured by stereotypes Expectations to change and conform
Individualisation as an autistic woman	Differentiating from cultural beliefs Integrated sense of self
Staying connected with society	Involuntary disconnection with others Meaningful connections Relational healing

### Theme 1: pervasive influence of socio-cultural values

Participants talked about a range of cultural values that they perceived to be prominent in both wider society and their own families. They drew attention to how cultural beliefs about different parts of their identity were learned through everyday interactions. Pressure to conform was experienced when they wanted to be or do something outside of cultural expectations.

#### Subtheme: power in the unsaid

Families were often the immediate cultural system where participants learned about what was valued in society. These values were often implicitly enacted within the scripts or the behaviours of the family. Lola, for example, reflected on a collection of behaviours that represented generations of her family’s values, such as:

Once you’re married, you stay married. You’re supposed to marry young . . . have produced kids, and everything was supposed to be overseen, sort of by God. (Lola)

This collection of behaviours spoke to overarching ideas about the institution of marriage and religion. Personal and family values clashed at times, with participants describing the tension that emerges from the often unexplored but strongly held stances from both sides. Lauren talked about how she would attempt to share her stance but anticipated a counter challenge from parents:

I have a strong belief that I will not have children . . . I sort of slip it into conversations, and my parents are like ‘No, no, you’ll change your mind’. (Lauren)

The implicitness in which attitudes were communicated in the wider social context left many participants feeling unsure of themselves or even vulnerable to exploitation. Sarah, felt that her school peers perceived her as ‘weird’ because of her differences in appearance, interests and character. However, this was communicated subtly over time through exclusionary behaviours:

They didn’t ever say it, but it was like they wouldn’t interact with me in the same way. (Sarah)

For Ambie, similar experiences and being bullied led to her constantly feeling apprehensive about potentially upsetting others, and Lola reflected on her vulnerability to mistreatment because of difficulties with gauging other’s intentions. There was a sense that social rules provided people with schemas in how to act in specific situations. However, these rules were often unspoken, and many participants found themselves operating blindly without this social knowledge that other people seemed to share:

I feel like there’s an expected kind of social etiquette that you have to follow . . . like the unwritten social rules of society . . . people are brought up into, and you’re kind of expected to . . . adhere to them, but without being told, really, what they are. (Emma)

#### Subtheme: obscured by stereotypes

Some participants described their observations of the prevailing view in society that autism largely impacts men and is characterised by highly stereotyped behaviours. Such views are exemplified in the following quotes:

It’s only men that are autistic. (Trinity)

‘Oh, you’re not autistic because you have friends or you can make eye contact’, or . . . they’re like ‘you don’t seem autistic’. (Emma)

There was an emphasis on others expecting to be able to ‘see’ autism, be it behaviourally or by association with gender. A cultural stereotype that conflated autism with ID was also absorbed by some participants and interestingly left them with a sense of not recognising autism themselves when unable to connect with stereotyped ways of being. For example, Emma had the following perception of autism which she did not identify with and therefore had not considered that she may be autistic when trying to understand the difficulties she experienced:

People who struggle to speak, or like couldn’t move properly or like flap their hands a lot and stuff. (Emma)

Such stereotypes also generated stigma and underestimation of abilities for some participants. Lauren expressed feeling powerless and limited by views about autistic people being homogenously disabled, when she believed that her application to join the military was rejected based on her autism diagnosis. She gave up on appealing through feeling invisible behind her diagnosis:

You have to write a letter, and that letter isn’t you . . . they just see the disability for me . . . but if they met me, they wouldn’t see the disability. (Lauren)

Defying cultural presumptions of what autism *looks* like, some women had to explain and, even defend their invisible needs. Rebecca was an academically and professionally accomplished woman. However, some social aspects of her job challenged her in ways that were unobservable to her colleagues. Rebecca’s status of being both competent and having needs was resolved in others by perceiving her imperceptible needs as exaggerations – shifting the difficulties she raised with her cultural environment, onto her, as character faults. When her repeated requests for reasonable adjustments were disregarded, she realised that some people perceived her as:

‘Being dramatic or making a fuss for no reason . . .’. or thought that ‘. . . she’d be fine, she’s . . . not making it up, but she’s making it seem a lot worse than it is’. (Rebecca)

Most of the participants thought that knowledge and understanding of autistic women have improved overtime in society; however, there was still progress to be made. Many participants took issue with the superficiality with which society talks about accepting differences, which perpetuate the invisibility of some autistic women:

. . . all that campaign . . . ‘Oh, it’s okay to be different’. But I feel like it comes with like hidden terms and conditions. Like ‘it’s okay to be different if we can meet your support needs’. Or ‘it’s okay to be different if we’re able to put in those accommodations’. Or ‘if you’re different in this way, but not in that way’. Or ‘if, you’re not too different’. (Emma)

#### Subtheme: expectations to change and conform

All women talked to some degree about pressure to change and conform with societal expectations. Interestingly, some women described the paradox they felt, such as Marion being advised to *be herself*, where her interpretation of this given wider experiences was:

I think what they were actually really saying is – change – and be something that we can handle, please. (Marion)

This pressure to change was also packaged for many women within expectations to be ‘quiet’, ‘good’ and ‘well behaved’, to be ‘polite’ and have good manners. These were juxtaposed with ‘bad’ or ‘rude’ behaviours that were deemed unacceptable. The assimilation of morality, along with rewards, reprimands and sometimes the removal of conflict, demonstrated the reinforcement mechanisms involved with shaping behaviours to the accepted norm from a young age.

The origins of such expectations to be agreeable for most participants could be understood from the gendered narratives about women in wider society. Lola and Sarah observed from school age that,

Girls were socialised to be a bit more careful with who they were. (Lola)

Women are meant to be like, nice, fluffy, everything. (Sarah)

Sarah had deduced that part of her struggles to fit in with peers at school was because her interests deviated from those descriptors. This is also highlighted in Rebecca’s reflection of how she was treated compared to her male colleagues within a male-dominated profession, emphasising a prolonged struggle to integrate:

What I find is that, I am perceived to be rude a lot more quickly than my male counterparts would be. So, I could say the same thing, but if I’m saying it as bluntly as they’re saying it, then I’m perceived as being rude. (Rebecca)

After thoroughly thinking through specific incidences independently and with others, Rebecca concluded that it was unlikely that she had behaved rudely. Nonetheless, she adapted her behaviour by ‘treading’ more carefully in the way she worded or suggested things to her colleagues to reduce confrontations at work. Her decision highlighted the tension between the necessity to adapt to her environment while holding onto her own values.

### Theme 2: individualisation as an autistic woman

Participants shared how they broke away from cultural beliefs that did not fit in with how they saw themselves or the world. This provided the necessary space for them to embark on journeys of self-discovery and develop narratives that felt more authentic and coherent to their self-perception.

#### Subtheme: differentiating from cultural beliefs

For many women, the process of separating themselves from expectations and beliefs that did not fit in with how they saw themselves was part of the journey to understanding their experiences. Ambie, Sarah and Marion spoke about how they moved away from beliefs held in their families that felt harmful to their wellbeing. While Lola, Lauren and Emma distinguished themselves from cultural ideas relating to disability and autism. These involved self-reflection, social analysis and experimentation to make room for their own views.

Trinity externalised demoralising beliefs about herself as fundamentally ‘bad’. These developed from earlier experiences of reacting aggressively to distress that left her feeling ashamed. Through the Internet and movies, Trinity developed a more nuanced understanding of emotions and experiences in autistic and non-autistic people. She started to understand the difference between ‘bad behaviour’ as deemed by societal norms versus needs relating to autism:

Oh, so that wasn’t bad behaviour. It was a sensory overload. (Trinity)

Trinity did not absolve herself from the responsibility of her actions and continually worked at managing her sensory overloads. However, the shift in her self-perception empowered her to make the decision to study at university and develop meaningful friendships.

#### Subtheme: integrated sense of self

Recognising that they were autistic was sometimes the missing piece of knowledge that participants needed to make sense of experiences that felt insufficiently explained by factors such as trauma or anxiety:

I identify with the autism; I identify with the mental health as well, but with the autism, it’s me . . . when I was about my late 30s, I kept thinking . . . there’s something not right. Why do I keep biting myself and hitting things? And why do I get angry so quickly? And why do I do this? Why do I get that? And why do I have, them call them tantrums, but they’re not tantrums, they’re meltdowns? (Ambie)

In the process of self-discovery, some women talked about unearthing abilities that they were not initially aware of, such as studying (Lauren), studying online (Marion), and poetry (Ambie), leadership (Lola) and media skills (Trinity). Some women talked about establishing their own values and making their own decisions amid the weight of expectations from others, such as in their choice of career (Rebecca and Emma) and interests (Sarah and Rebecca). For others, it was a process of developing a more balanced perspective of themselves:

I count myself as an ambivert . . . when I’m with my friends, quite an outgoing loud person . . . and that’s something I’ve had to hide a lot. I think growing up, parents always saw me as an introvert. Everyone told me I was an introvert. But now, it’s very obvious I’m not fully an introvert. I quite like being an introvert sometimes, but it’s that culture of having to adapt to different situations. I’m a very different person with, around my family as well. And that culture is different from what I have had to experience at university. (Lauren)

This drawing together of new and old identities was also experienced by other participants and emphasised how Lauren formed a richer narrative of herself by integrating aspects of who she was with how she is. There is also an agency that emerges with participants who talk of a more multidimensional self-image:

I kind of have a lot of different, I don’t want to call them personalities, they’re not big enough to be personalities. I have a lot of different, roles, that I pick depending on the context of where I am, what I’m doing at the time, who I’m speaking to. So, if I was, completely left to my own devices . . . I probably wouldn’t say much to anyone at all. I certainly wouldn’t do things like, be sitting here or going to work. Or, if I did, I would not be as productive. (Rebecca)

The autistic women in this study were vibrantly diverse in terms of backgrounds, personalities and strengths. They also shared different life events that have contributed to their character, such as sexual, emotional, and physical abuse, bereavement, physical impairments and relational traumas. It was hard to delineate where autism ends and other identities start, but being autistic was not the sole marker of their identities:

I don’t think you can separate [being autistic and being me] out really, being autistic is about your behaviours, well the behaviours and traits are you, so, it’s really, I’m not saying that your whole identity is being autistic, but they’re kind of meshed in, so when someone, says ‘Oh do you do that because you’re autistic?’ Er, yes and no. I do that because I do that. (Marion)

### Theme 3: staying connected with society

All participants shared experiences of isolation and disconnection in their lives that were involuntary. There was a clear longing for meaningful connections with people that also had a restorative impact for most participants who have experienced marginalisation.

#### Subtheme: involuntary disconnection with others

Experiences of feeling misunderstood have been interspersed across the superordinate themes for all participants, such as being misperceived as discourteous or unintelligent. These experiences have left many of the participants feeling hurt, alone and disconnected as described by Ambie:

Sometimes when I go somewhere, if I have a misunderstanding with someone, I find it hard to connect with them again . . . and sometimes if that happens, I feel so isolated. Because, you see, I’ve taken me with me, you know, I’m taking the damaged part away on holiday with me, which is quite challenging. (Ambie)

Ambie referred to the integrated nature of her mental health difficulties and experiences of sensory overload that were as much a part of her, as her creative and personable qualities. Hence, feeling misunderstood for how she might behave, for example, in reaction to feeling overwhelmed, has left her feeling vulnerable to personal rejection. Experiences of marginalisation were not uncommon for participants, and they often came with the sentiment of ‘what have I done now? Who have I offended?’. These ruminations often carried the duality of feeling accountable for relational disconnections and not knowing why.

There was a distinction between needing or enjoying time alone and experiences of disengaging from involuntary socialising that were driven by cultural expectations. This was apparent for many participants who socially withdrew during their childhood, but started to appreciate socialising in adulthood:

There was huge pressure from my parents to, make this friend . . . and they would introduce a lot of random people to me, and try and make them befriend me. And that kind of never really worked out. So there was an expectation going to a lot of social events, social clubs and things like, I was a Rainbow . . . Brownie . . . Guide, and I hated all three of them and I dropped out of all three of them. But every time I hit a new age bracket, I was taken back to do the next one, to try and socialise. (Lola)

Lola’s experiences highlighted how one can simultaneously have physical proximity with people and feel relationally disconnected.

#### Subtheme: meaningful connections

It was evident that all participants wanted meaningful connections with other people. Most participants favoured quality over quantity of connections. Marion expressed the depth of interaction she sought:

. . . talking about, you know, life and what people feel about things and, sort of deeper conversations. (Marion)

Her sincerity towards getting to know others mirrored her own longing, which she articulated as a desire:

. . . to be understood . . . if there was somebody who understood me, then that was fantastic! (Marion)

Interests were often an important subculture that provided participants with the opportunity to connect with others who recognised them for something they chose to be. Trinity detailed the benefits of meeting others with the same interest as her, because:

. . . they don’t care if I’m autistic, we just have a laugh . . . we talk about anything. (Trinity)

Her statement hints at the stigmatised view of autism within the wider culture and how mutual interests can remind her and others of their shared humanity.

For Lauren, she discovered her enjoyment for socialising once it became meaningful for her. Lauren’s parents managed her social calendar when she was younger. She mostly played a passive role in socialising, and sometimes actively avoided it. When Lauren gained relative autonomy and independence at university, she was able to engage and enjoy being with others:

I will also count one of my hobbies as socialising . . . When I came to university, I was getting all into the societies . . . I was doing, all these things that I had to do myself. That was different because it was always my parents, I did girl guiding for 13 years, and that was just something that was expected of me because my parents would take me to them. And now it’s something, it just felt something different, to be able to do it myself and be able to socialise. (Lauren)

#### Subtheme: relational healing

The restorative impact of supportive and sincere relationships was apparent for all participants – from the formation of new relationships, to rebuilding existing bonds with families. Furthermore, women described embracing themselves for who they are and wanting to enjoy better relations with family as well as broader people in social encounters.

Lauren had built up a lot of walls for surviving as a child and described how her adoptive parents spent 10 years trying to knock them down. A combination of family therapy and newfound independence at university helped Lauren communicate her needs and build relationships. Lauren explained how she had created personas as a child because she did not believe that she was a ‘generally likable person’, and through learning to communicate her vulnerabilities rather than ‘pretend that everything was normal’ helped her realise that she should be herself. Lauren’s earlier beliefs about herself were formed in relation to her experiences with her family of origin, as well as feeling out of place with her adoptive family who had a very different culture in terms of socio-economic background, geography and religion. It was therefore unsurprising that for Lauren to learn to appreciate her own ‘wacky’ character, it also involved relating differently with the significant people around her.

The healing effect of relationships was bi-directional. In supporting others, many participants saw themselves as someone with the resources to bring about positive change for others. This ranged from Trinity supporting her boyfriend through bereavement to Marion building a positive self-perception for her autistic child; through to Ambie raising large sums of money through her poetry performances for the autism charity that has supported her. Emma resolutely talked about choosing her profession:

. . . because, I met some amazing nurses when I was in hospital. They really helped me, so I wanted to be able to help other people. (Emma)

Emma’s intension to give others the same compassion she had received when she needed it, showed her shift from being someone who needed help to someone who could help others.

## Discussion

This qualitative study aimed to explore the impact of culture on the lives of autistic women based in England. Overall, the study highlighted the complexity and dynamic nature of interaction between autistic women and their socio-cultural context. The findings elucidate a range of challenges experienced by women in managing societal expectations, specifically those based on gender and autism stereotypes. The women in this study experienced pressure to conform to gender roles, and interpretations of their behaviours were also affected by the invisibility of their autism. Separating from unhelpful stigmatising interpretations about themselves and developing a different relationship with their cultural contexts helped with a more integrated sense of self.

Deviating from cultural expectations has been described as a challenge in broader literature, including for women who identify with other minoritised groups. For example, LaVaccare et al. (2018) found that women who identify as lesbian or bisexual experienced difficulties with healthcare providers attributed to normative assumptions about sexual orientation. For women with autism, the double paradox appears to be the additional deviation from narrow and male-dominated view of autism itself.

Gendered socialisation played a key role in the experiences of women in this study, especially in the form of cultural pressures to get married, have children and behave in a stereotypically feminine way. Non-conformity was often met with coercion to change, bullying or marginalisation. The impact of which included obstacles for identifying autism, seeking relevant support and developing a more coherent self-concept. These findings reflect existing research on the difficulties of autistic women who did not fit in with conventional constructions of being a *woman* or indeed *autistic* (Bargiela et al., 2016; Kanfiszer et al., 2017; Tint & Weiss, 2018). Research that has compared gendered expectations for agreeableness and compliance found that women were expected to be more agreeable and cooperative to requests. In addition, women were judged more harshly if they were perceived to hesitate with helping another female (Roberts & Norris, 2016). These gendered expectations manifested in autistic women’s lives too and often impacted on peoples’ acceptance of individuality and for some, on their own alignment of internal sense of abilities and external effort to develop coping methods. Such patterns of responding have also been observed by Tint and Weiss (2018) who described camouflaging by autistic women to fit the female social landscape.

Tension between autistic women and their cultural contexts was frequently related to balancing their connectedness with others and establishing their own identity. We see this in the camouflaging behaviours of autistic people who try to assimilate at the cost of their sense of authenticity (Hull et al., 2017). Autistic women have also repeatedly expressed a desire to forge meaningful identities (Bargiela et al., 2016; Kanfiszer et al., 2017; Leedham et al., 2020; Webster & Garvis, 2017), and many autistic people want ‘to be able to define themselves on their own terms’ (Milton & Sims, 2016, p. 527). For the autistic women in this study, this was further complicated by the implicit nature of cultural values. There was a sense of first needing to be able to identify biased perceptions before they could differentiate or integrate into their identities.

Positive relationships aided participants to embrace their own individuality and vice versa, greater self-acceptance also improved relationships with existing and new people in autistic women’s lives. Consistent with identity theories that see identity formation as involving both internal and external cultural constructions (Duveen, 2013), the self-concept of participants was affected by the way their social groups perceived and interacted with them (Tajfel & Turner, 1979). In line with theories on the effects of marginal social identities, autistic women resolved dissonance between self and cultural perceptions by making either internal or external changes (Breakwell, 1978). Readjustments to self-perception could be both disempowering and empowering, such as when study participants saw themselves as deficient in some way or when they tried to instigate changes to external perception through the written word, speaking with friends, family and communities. Similar changes in self-view could also be seen in lived-experience research of autistic women who questioned their gender identity when they differed from normative representations of womanhood (Kanfiszer et al., 2017) or saw themselves as positive agents of change to others (Webster & Garvis, 2017).

Goldman (2013) highlighted the importance of better understanding the interaction of gender-based socialisation with autism, and our findings contribute multiple insights into this intersection. Clinicians and professionals working in settings such as diagnostic clinics, mental health services and schools should be informed about the way in which culture may result in biases towards autistic women and the far-reaching consequences of this for their sense of self. For example, caution should be exercised in placing an unequal amount of responsibility on autistic women to behave in line with convention, which may negatively impact their wellbeing (Hull et al., 2017). Clinical formulations and interventions could attend more to the cultural values of relevant identity markers such as autism and gender. This can provide important information about how autistic women have negotiated belonging and individuality (Milton & Sims, 2016), along with how they have adaptively and maladaptively coped with the potential dissonance with their cultural environment. Supporting autistic women to make sense of these complex experiences can help to prevent the harms of stigmatised narratives about being explicitly and implicitly different.

To our knowledge, this is the first study that has explicitly examined the reciprocal relationship between autistic women and their socio-cultural contexts. On the surface, the sample appears relatively homogeneous in relation to aspects such as the majority being of White ethnic heritage and having no religious affiliations. However, there were variations in terms of family composition and socio-demographic background that shaped their experiences. The research also supports the use of IPA as a research method with autistic people. IPA offers an opportunity to address power relations between researcher and participants, while engaging with an in-depth exploration of autistic women’s perspectives (MacLeod, 2019). Nonetheless, there are some caveats when interpreting the findings. Given that the women in this study mainly identified with a White ethnicity, less can be extrapolated to the impact of culture on autistic women who may be negotiating different cultural norms and may identify with multiple minoritised characteristics. The relationship with culture may also differ for women diagnosed in childhood compared to those diagnosed in adulthood. This was not considered within the study. Furthermore, although the premise of the study was to elucidate an understanding of the intersection of gender and culture for autistic women specifically, future research would benefit from comparing and contrasting the narratives of women and men. This will help deepen awareness of biases that help and hinder being on one’s own terms.

## Conclusion

This study examined the inter-relatedness between autistic women and their social and cultural contexts. The findings highlighted the pervasive influence of cultural values on different aspects of autistic women’s lives. Participants also reflected on the impact of their immediate and wider cultures on their lived experiences and self-perception, underscored by the desire to stay connected with society and the positive impact of belonging. A shift towards a more contextualised understanding of autistic women is arguably necessary for moving away from a stigmatised perspective of being both a woman and autistic. This will aid recognition of autistic women socially and clinically. A joint relational responsibility between autistic and non-autistic people should be reflected in clinical formulations that inform support and research agendas.
